# Health Care Expenditure and GDP in African Countries: Evidence from Semiparametric Estimation with Panel Data

**DOI:** 10.1155/2014/905747

**Published:** 2014-03-06

**Authors:** Zhike Lv, Huiming Zhu

**Affiliations:** School of Business Administration, Hunan University, Changsha 410082, China

## Abstract

A large body of literature studies on the relationship between health care expenditure (HCE) and GDP have been analyzed using data intensively from developed countries, but little is known for other regions. This paper considers a semiparametric panel data analysis for the study of the relationship between per capita HCE and per capita GDP for 42 African countries over the period 1995–2009. We found that infant mortality rate per 1,000 live births has a negative effect on per capita HCE, while the proportion of the population aged 65 is statistically insignificant in African countries. Furthermore, we found that the income elasticity is not constant but varies with income level, and health care is a necessity rather than a luxury for African countries.

## 1. Introduction

It is important for policymakers to know the relationship between health care expenditure and income, knowing this relationship helps them to make wise judgments, plan health reforms, and allocate resources efficiently.

It is generally believed that there is a strong and positive relationship between health care expenditure and income, which is well established in the literature. However, there is no consensus on whether the income elasticity of health expenditure is greater or less than 1, Baltagi and Moscone [1] and Santiago et al. [2] provide excellent overview of it. The income elasticity of health expenditure can be defined as the percentage change in health expenditures in response to a given percentage change in income. If the elasticity is less than one, health care will be classified as a “necessary” good; that is, health expenditures increase more slowly than income. While if the elasticity is greater than 1, then health care will be defined as “luxury” good.

There is already a substantial literature on studying the link between HCE and GDP, but almost without exception, these studies have been limited to OECD countries or developed countries, and we will review this literature in the following section. As it can be seen, most of previous studies have been performed at OECD countries. Very little literature has been done for African countries, this maybe because of data availability. To the best of our knowledge, Gbesemete and Gerdtham [3] and Jaunky and Khadaroo [4] are the only two studies in the literature that examine this link in African countries. And this paper seeks to contribute to filling this gap by exploring panel data from 42 African countries during the period 1995–2009.

Compared with previous studies, this study contributes to the literature in the following aspects. First, as we all know, most of early studies use parametric techniques that assume a functional form, like a linear one. In fact, this may be unavoidable to obtain the estimator inconsistent, if their true relationship is nonlinear [5–7]. Therefore, the adoption of a semiparametric partially linear panel data approach has the advantage that it does not impose a specific functional form on the relationship between HCE and GDP. Second, Crémieux et al. [8] find that lower health care expenditures is associated with significantly higher infant mortality rate, and Gupta et al. [9] show some evidences that health expenditure reduce childhood mortality. In addition, considering the fact that the infant mortality rate (IMR) is relatively higher in Africa than other regions, we consider this variable (IMR) in our model. Finally, we reconsider the relationship between income and health expenditure for countries at different levels of development.

The remainder of this paper is organized as follows. The next section reviews the literature.  Section 3 presents the data description and econometric model.  Section 4 discusses estimation results. Finally, Section 5 draws up policy recommendation and concludes the paper.

## 2. Literature Review

In this section, we review the existing papers and their main result about the income elasticity of health care expenditure. Since the seminal paper by Newhouse [10], who observed that over 90 percent of the variation between countries in per capita health care expenditure could be explained by variations in per capita GDP, it has become popular to investigate whether the income elasticity of health care expenditure is more or less than 1. Throughout the existing research studies, Past researches in this field may be classified into three results, one found the elasticity was higher than 1 [11–14] and others believed that the elasticity was less than 1 [1, 4, 15–17], still others obtained a result that the elasticity was around one [2, 3, 18, 19]. The discrepancy of income elasticity in this literature could be attributed to a variety of reasons like using different econometric methods, different data, and explanatory variables.

Early studies on this topic usually used a single cross-section data, Parkin et al. [20] found that the income elasticity was 0.90 using cross-section data for 18 OECD countries. While Gerdtham et al. [21] estimated an income elasticity of 1.33 using cross-section data for 19 OECD countries. Later on, some researchers doubt their results because using a single year's cross-sectional data ignored the presence of unobservable country specific effects and also may exhibit other variables playing a key role to influence the result. To remedy these drawbacks, researchers have used panel data model or time series data model to analyze the relationship between HCE and GDP [18, 22, 23]; they also used a richer set of explanatory variables, such as the percentage of the population over the age 65, the percentage of the population under 15, and the proportion of HCE that is publicly funded [13, 24, 25]. But still they do not reach a consensus whether the income elasticity is larger than 1 or less than 1. More recently, much attention has been focused on the question whether health care and GDP are stationary or not. Hansen and King [22] studied the time series from each separately and found that one can only rarely reject the unit root hypothesis for either GDP or HCE. McCoskey and Selden [26] revisited the question of unit roots in the OECD data proposed by Hansen and King [22], and they rejected the presence of unit roots for HCE and GDP by using the IPS test. Baltagi and Moscone [1] considered the nonstationary and cointegration properties between HCE and income; their results demonstrated that health care was a necessity rather than a luxury. This result is consistent with previous studies [27], and these authors conclude that income elasticity is less than 1. However, it is well known that the parametric models may be misspecified, and estimators obtained from misspecified models are often inconsistent. To remedy this shortcoming, more and more researchers are starting to use some nonparametric approaches to study the income elasticity of health care expenditure. One of the key features of the nonparametric approach is that little prior restriction is imposed on the model's structure. By testing for structural breaks in panel varying coefficient model, Liu et al. [13] found that the relationship between HCE and GDP was highly nonlinear and the income elasticity was not constant but it varies with income level.

## 3. Data and Method

### 3.1. Data Description

The data structure is a balanced panel of 42 African countries during the period from 1995 to 2009. Some countries are excluded from this study, because it is not possible to obtain detailed corresponding statistics. Our data are gathered from the World Bank's World Development indicators 2012. The World Bank classified countries into four categories (i.e., low income, low-middle income, upper-middle income, and high income) by GNI. In this paper, we put the low-middle income and upper-middle income as middle income group. So, these 42 African countries include 22 lower income countries and 20 middle income countries. The dependent variable used in this paper is total health care expenditure per capita measured in US dollars in real prices, adjusted for purchasing power parities (PPP). To fully examine the relationship between health care expenditure and income, we include income and two nonincome variables as explanatory variables. The income is replaced by per capita GDP, The two nonincome variables are defined as follows: infant mortality rate per 1,000 live births and the percentage of population age 65 and above (POP65). All these variables are expressed in logarithm.  Table 1 shows the basic statistics.

### 3.2. Econometric Model

To study the link between per capita HCE and per capita GDP, the conventional approaches assume that their relationship is linear, and the most commonly used parametric specification is the following one:

(1) $$\begin{array}{c} {\text{lnhce}}_{it}=\mu_{i}+\gamma_{1}{\text{lngdp}}_{it}+\varepsilon_{it}, \end{array}$$

where lnhce_*it*_ is logarithm of HCE per capita of country *i* at year *t*, lngdp_*it*_ is logarithm of GDP per capita of country *i* at year *t*, and *μ*_*i*_ is the country fixed effects. Moreover, additional explanatory variables, like the percentage of population age 65 and above (POP65), infant mortality rate per 1,000 live births (IMR), denoted by a *d* × 1 vector of *z*_*it*_, can be added to yield

(2) $$\begin{array}{c} {\text{lnhce}}_{it}=\mu_{i}+\gamma_{1}{\text{lngdp}}_{it}+z_{it}^{T}\gamma_{2}+\varepsilon_{it}. \end{array}$$

Just as Yatchew [28] argues, most economic theories do not identify a specific functional form for the link between a dependent variable and the explanatory variables in a regression. In addition, Mehrara et al. [12] found the relationship between income and health expenditure is nonlinear by using panel smooth transition regression. Therefore, to avoid possible model misspecifications of the parametric form, we use a nonparametric form, and the simplest nonparametric specification is

(3) $$\begin{array}{c} {\text{lnhce}}_{it}=m\left({\text{lngdp}}_{it}\right)+\mu_{i}+\varepsilon_{it}, \end{array}$$

where *m*(·) is an unknown smooth function, and *ε*_*it*_ is assumed to be i.i.d. with a zero mean, finite variance. Like the parametric cases, we include some other variables in the nonparametric panel data model:

(4) $$\begin{array}{c} {\text{lnhce}}_{it}=m\left({\text{lngdp}}_{it},z_{it}\right)+\mu_{i}+\varepsilon_{it}. \end{array}$$

The above model may consider the relationship between HCE and GDP more accurate, however, as is known to all, high dimensional nonparametric model suffers from the dimensionality curse. To remedy this shortcoming, one resolution is to follow Baltagi and Li [29]. Let *y*_*it*_ = lnhce_*it*_ and *x*_*it*_ = lngdp_*it*_, then we can specify a partially linear panel data model as follows:

(5) $$\begin{array}{c} y_{it}=m\left(x_{it}\right)+z_{it}^{T}\gamma +\mu_{i}+\varepsilon_{it}, \end{array}$$

where only lnhce_*it*_ enters the unknown function *m*(·) and the other variables are specified parametrically. Our model permits the inclusion of other explanatory variables without suffering the curse of dimensionality. To remove the fixed effects, motivated by the first differencing method for linear panel data models, one can consider the following first-order difference model:

(6) yit−yi,t−1 =m(xit)−m(xi,t−1)+(zit−zi,t−1)Tγ+εit−εi,t−1.

As for Model (6), Baltagi and Li [29] proposed to approximate [*m*(*x*_*it*_) − *m*(*x*_*i*,*t*−1_)] by the following series differences:

(7) $$\begin{array}{c} p^{k}\left(x_{it},x_{i,t-1}\right)=\left[p^{k}\left(x_{it}\right)-p^{k}\left(x_{i,t-1}\right)\right], \end{array}$$

where *p* ^*k*^(*x*) are the first *k* terms of a sequence of functions (*p*_1_(*x*), *p*_2_( ),…). Then, (6) boils down to

(8) yit−yi,t−1 =(pk(xit)−pk(xi,t−1))θ+(zit−zi,t−1)Tγ+εit−εi,t−1.

Once parameters $\hat{\theta }$ and $\hat{\gamma }$ have been estimated, the values of ${\hat{\mu }}_{i}$ can be calculated to recover the error component residual [30].

(9) $$\begin{array}{c} {\hat{u}}_{it}=y_{it}-z_{it}^{T}\hat{\gamma }-{\hat{\mu }}_{i}=m\left(x_{it}\right)+\varepsilon_{it}. \end{array}$$

Now, Model (9) is a nonparametric panel data model, we can use kernel smoothing method like spline regression [30], Nadaraya [31], and local linear [32]. In our empirical analysis, to estimate *m*′(·), where *m*′(·) stands for the derivative of the function *m*(·), we adopt the conventional local linear approach. *m*′(·) can be estimated by solving the following optimization problem:

(10) $$\begin{array}{c} \underset{a,b}{\min}\sum_{i=1}^{N}\sum_{t=1}^{T}{\left[{\hat{u}}_{it}-a-b^{T}\left(x_{it}-v\right)\right]}^{2}\times k_{h}\left(x_{it}-v\right), \end{array}$$

where *k*_*h*_(·) is invariable kernel function satisfying the usual regularity conditions, *h* is the bandwidths (or smoothing parameters), and (*a*, *b*) = (*m*(·), *m*′(·)). For simplicity, we set $U={\left[{\hat{u}}_{it}\right]}_{NT\times 1}$, Γ = [1, (*x*_*it*_ − *v*)^*T*^] and *e* = (0,1)^*T*^, then the local linear estimator of *m*′(·) is given by

(11) $$\begin{array}{c} {\hat{m}}^{\prime }\left(u\right){=e\left[\Gamma^{T}k_{h}\left(x_{it}-v\right)\Gamma \right]}^{-1}\Gamma^{T}k_{h}\left(x_{it}-v\right)U. \end{array}$$

As we all know, compared to the selection of the bandwidths, the choice of the kernel is less important for the properties of the resulting estimator. So, in this paper, we use standard univariate Gaussian kernel, and we use the least squares cross-validation method to select the bandwidths *h*.

## 4. Estimation Results

In this section, we firstly present the results from the parametric panel data mode (2). Hausman test (*P* = 0.0000) supports the fixed effect model against the random effect model. Columns 1-2 of Table 2 report the parametric estimation results. Note that a quadratic polynomial function is still significant. This casts doubts on the conventional linear specification.

**Figure 1:**
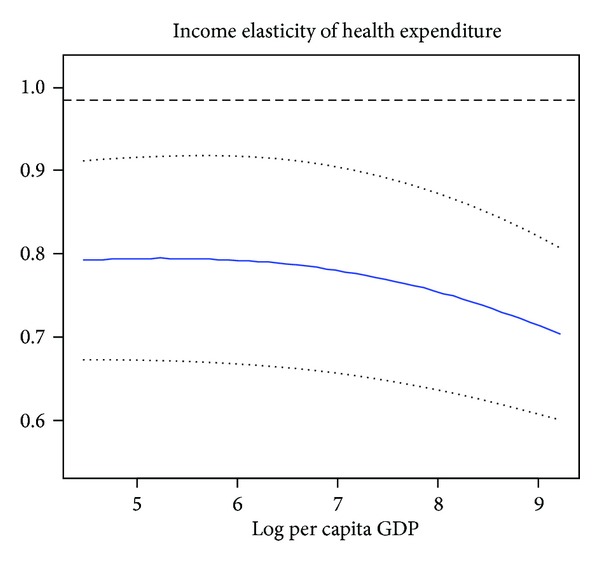
The income elasticity estimator of all African countries, the solid curves represents *m*′(lnGDP).The dotted curves correspond to its 95% confidence interval.  The level dashed line represents the estimate from the linear model.

To estimate the semiparametric partially linear panel data model, we use the proposed methodology which is described in above section. First, we estimate all 42 African countries. In the last column of Table 2, we report the estimator results for the linear part and their corresponding standard errors. From Column (3) of Table 2, we find that POP65 coefficient is statistically insignificant in these 42 African countries, and this finding can be interpreted as per capita HCE is not affected by POP65. While IMR is found statistically significant in African countries, this means that a change in infant mortality rate has a negative effect on health expenditures. IMR is one of the most important indicators to show a country's social condition. As we all know, due to various causes of morbidity like malaria, respiratory tract infections, HIV/AIDS, and malnutrition in Africa, most of African countries suffer from relatively high IMR, which is associated with lower health care spending. Lower health care spending increases IMR, and in turn, higher IMR decreases health care spending (vicious cycle). Moreover, by observing Figure 1, we find that the income elasticity is relatively flat, and the income elasticity is between 0.71 and 0.78, this means that health care is “necessary” good rather than “luxury” good in African countries.

**Figure 2:**
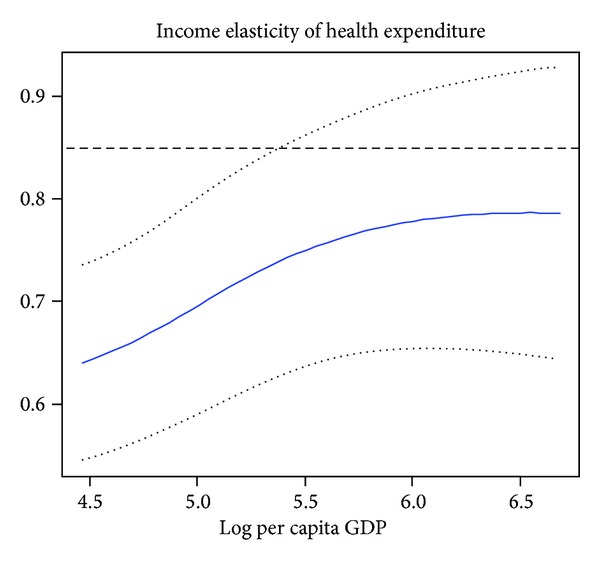
The income elasticity estimator of lower income African countries, the solid curves represents *m*′(lnGDP).  The dotted curves correspond to its 95% confidence interval.  The level dashed line represents the estimate from the linear model.

**Figure 3:**
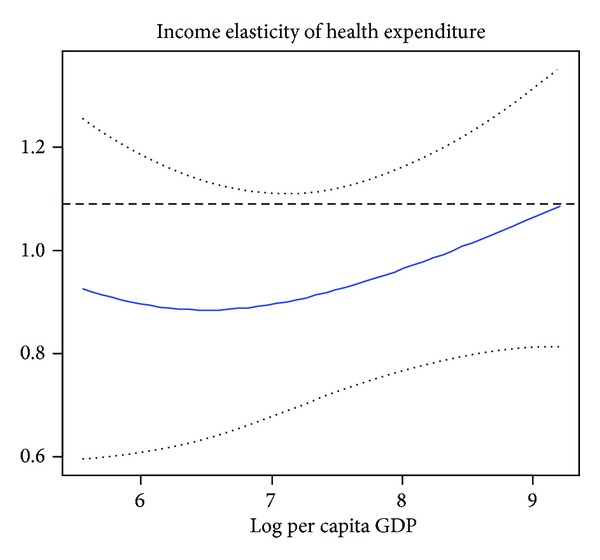
The income elasticity estimator of middle income African countries, the solid curves represents *m*′(lnGDP).  The dotted curves correspond to its 95% confidence interval. The level dashed line represents the estimate from the linear model.

Second, to study the relationship between income and health expenditure for countries at different levels of development, we use the World Bank classification system to put these 42 African countries into different country income groups. In column (2) and column (3) of Table 3, we present the estimator results for lower income countries and middle income countries, respectively. According to Table 3, we find that POP65 is still statistically insignificant for middle income countries or lower income countries; we believe that the main reasons are that the poverty and disease lead many young men to death. According to World Bank's World report, life expectancy at birth in Sub-Saharan African (all income levels) is 54 in 2010. In addition, for reasons of history, natural condition as well as system in these countries, health care is just for rich people; most patients in African countries are too poor to pay fees. Especially in some poor countries, providing even the most basic levels of health care for their people is out of the question. IMR is found to be statistically significant both for lower income and middle income countries; the negative coefficient means that an increase in infant mortality rate would reduce health care expenditure. Moreover, our result shows that the coefficient of IMR is higher for lower income countries than for middle income countries. A 1% increase in IMR was associated with 0.9653% reduction in health care health spending in low income countries and 0.7290% reduction in middle income countries. Figures 2 and 3 illustrate the semiparametric estimation of income elasticity in lower income countries and middle income countries, respectively, where lower and upper bounds of 95% confidence intervals are also drafted. Based on Figures 2 and 3, we can find that the income elasticity for health care is relatively higher for middle income countries than lower income countries, the middle income group shows higher responsiveness to increases in income. Our finding is consistent with Farag et al.'s [17] study; they obtain that the income elasticity for health care is highest for middle income countries and lowest for low income countries, with high income countries falling in the middle. From Figures 2 and 3, we can observe that the income elasticity is less than one in lower income African countries, while the income elasticity is close to unity in middle income African countries.

## 5. Policy Recommendation and Conclusions

In this paper, we examined the relationship between per capita health care expenditure and per capita GDP in 42 African countries during the period 1995–2009. Our paper has several advantages. First, in research methods, we applied a semiparametric partially linear model. On the one hand, this method requires no or very limited assumptions to be made about the format of the data, and it is also very useful for dealing with unexpected, outlying observations that might be problematic with a parametric approach. On the other hand, fully nonparametric methods may be unreliable due to the so-called “curse-of-dimensionality”. Therefore, a semiparametric partially linear panel data model is more preferable.

Next, we take into account the relationship between income growth and health spending growth for countries at different levels of development. Our findings indicate that IMR has a negative effect on health expenditures. However, we found that the percentage of older people has no positive effect on health expenditures in African countries.

Finally, our results suggest that the elasticity of income for health spending is varied by the level of per GDP, and health care is necessary good for African countries.

Based on our results, some practical suggestions are put forward to provide reference for policymakers. First, since IMR also play a key role as well as income to determine the level of HCE in African countries, an appropriate, robust, and sustainable model for improvement in health system performance is essential in order to reverse the declining trends in health and development status and break the vicious cycle of higher IMR and lower HCE in Africa. Second, our results suggest that POP65 is insignificant in African countries. Therefore, a fair health care system should be taken into account to improve life expectancy. Finally, as the income elasticity is a measure of the degree of responsiveness of health spending to changes in a country's income, our findings indicate that the income elasticity of health expending is less than one, so it is necessary for policymakers to take every aspect into consideration not only to manage the limited health funds in an effective way, but also to focus on increasing income.

## Figures and Tables

**Table 1:** Descriptive statistics (1995–2009, observations = 630).

Variable	Minimum	Mean	Maximum	Std-deviation
lnHCE	1.344	3.347	6.365	1.034
lnGDP	4.634	6.344	9.213	1.029
lnPOP65	0.637	1.128	1.936	0.245
lnIMR	1.411	4.238	5.071	0.619
22 lower income countries	Benin; Burkina Faso; Burundi; Central African Republic; Chad; Comoros; Congo, Dem.Rep; Eritrea; Ethiopia; Guinea; Guinea-Bissau; Kenya; Madagascar; Malawi; Mali; Mozambique; Niger; Rwanda; Sierra Leone; Tanzania; Togo; Uganda			
20 middle income countries	Angola; Algeria; Botswana; Cameroon; Congo, Rep.; Cote d'Ivoire; Djibouti; Egypt, Arab Rep.; Ghana; Gabon; Lesotho; Mauritania; Mauritius; Nigeria; Senegal; Sudan; South Africa; Swaziland; Tunisia; Zambia			

**Table 2:** Parametric estimation results.

Variable	Parametric model		Semiparametric model
	1	2	
Constant	−1.0293** (0.5296)	−0.1887 (0.8002)	
lnGDP	0.9882*** (0.0261)	0.7583*** (0.1662)	
lnGDP^2^		0.0177* (0.0104)	
lnIMR	−0.5525*** (0.0783)	−0.5563*** (0.0784)	−0.8574*** (0.2140)
lnPOP65	0.3895**(0.1672)	0.3074* (0.1770)	0.5156 (0.5521)
Country dummies	Yes	Yes	Yes
Year dummies	Yes	Yes	Yes
Adjusted *R*-square	0.7718	0.7710	0.4142

The dependent variable is lnHCE. Robust standard errors in parentheses.

*Significant at the 10% level.

**Significant at the 5% level.

***Significant at the 1% level.

**Table 3:** Semiparametric regression results: country income groups.

Explanatory variable	22 lower income countries	20 middle income countries
lnPOP65	0.4757 (0.9736)	0.5772 (0.6849)
lnIMR	−0.9653*** (0.3252)	−0.7290** (0.2938)
Adjusted *R*-square	0.3285	0.5108

The dependent variable is lnHCE. Robust standard errors in parentheses.

*Significant at the 10% level.

**Significant at the 5% level.

***Significant at the 1% level.
